# Supplementary material for: The Influence of Cohesin on the Short-Scale Dynamics of Intact and Damaged Chromatin in Different Phases of the Cell Cycle
Source: Int J Mol Sci. 2025 Sep 11;26(18):8837. doi: 10.3390/ijms26188837 (PMC12469696; doi:10.3390/ijms26188837)
Supplement: Supplementary file 1 [file ijms-26-08837-s001.zip › Supplementary figures and tables.pdf]

International Journal of *Molecular Sciences*

MDPI

Supplementary Figures and Tables

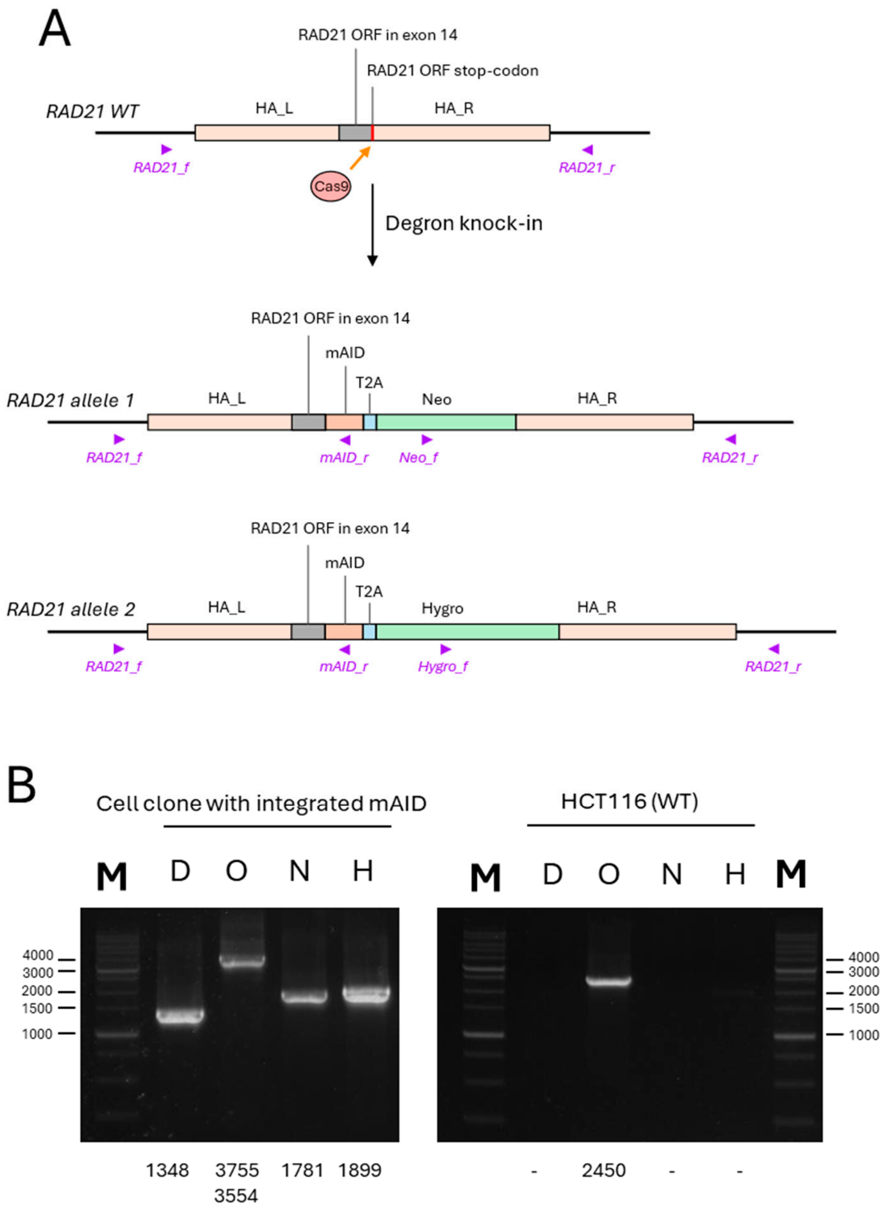

Figure S1. Integration of mAID degron into the RAD21 gene. (**A**) The scheme of mAID degron integration into the RAD21 gene, using CRISPR/Cas9-mediated knock-in. The positions of the homology arms (HA_L and HA_R) used for homologous recombination and the end of the RAD21 reading frame (in exon 14 of this gene) are marked. To select cells with homozygous integration, the cells were sequentially cultivated in the presence of two antibiotics (hygromycin B and G-418), resistance to which was conferred by the integrated constructs. (**B**) PCR analysis of construct integration into the genome of the cell clone, which was subsequently used to generate HCT116_RAD21_AID cells. The expected sizes of PCR products (in bp) are indicated below the lanes. HCT116(WT) cells were used as a negative control in PCR analysis. The primer pairs were designated as D (degron; RAD21_f/mAID_r), O (out; RAD21_f/r), N (Neo; Neo_f/RAD21_r), and H (Hygro; Hygro_f/RAD21_r). The positions of the primer binding sites are marked in Scheme A. Lanes M - DNA molecular length marker (DNA Ladder 1 kb, Evrogen).

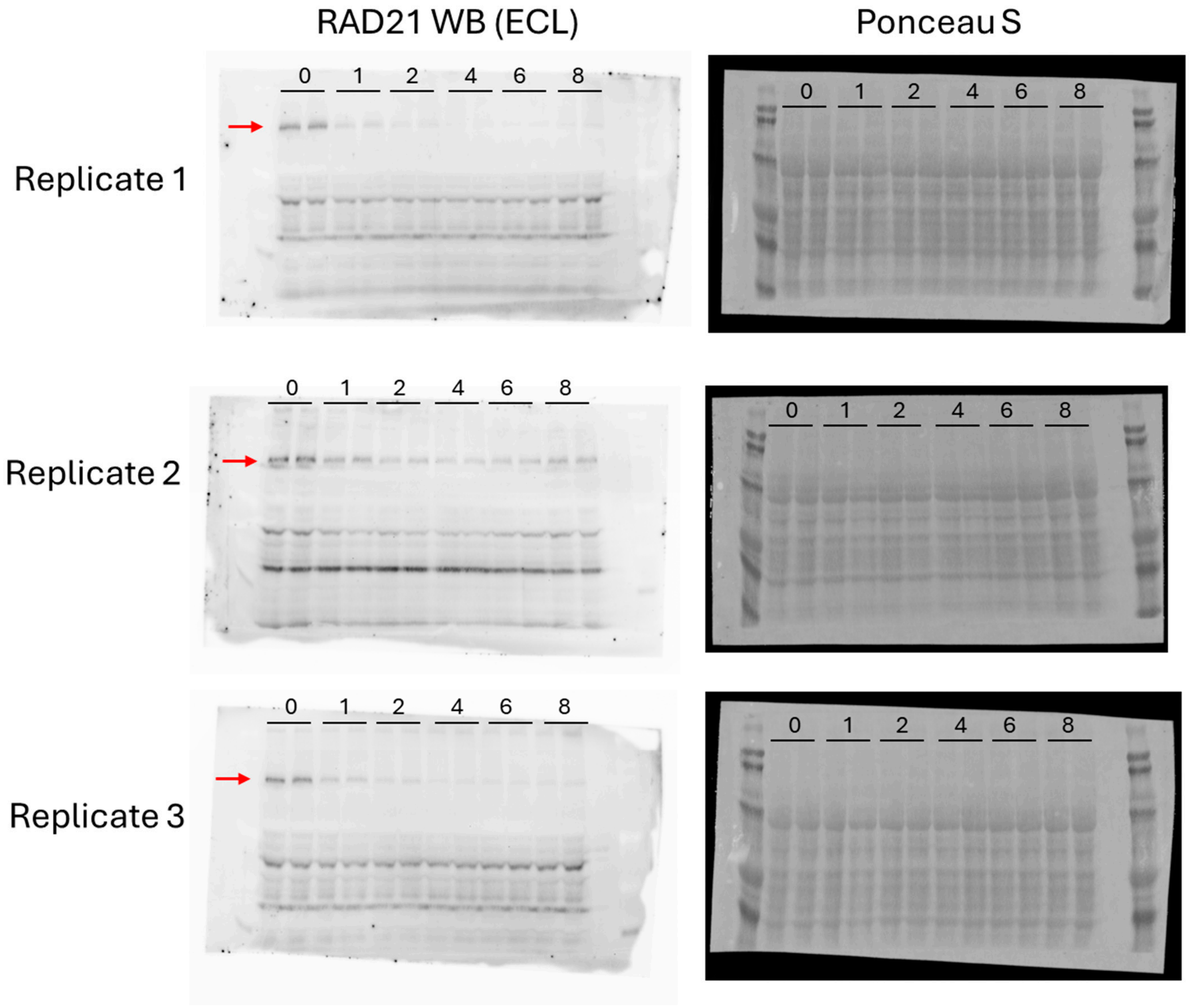

Figure S2. Raw membrane images showing the kinetics of RAD21 depletion in HCT116_RAD21_AID cells. An image of the membrane developed with ECL (left row) and an image of the membrane stained with Ponceau S to normalize for total protein (right row) are shown for each biological replicate. The loaded samples corresponding to the indicated hours of incubation with auxins are labeled above the lanes. Each sample was loaded in two technical replicates to improve the accuracy of the analysis. The arrow marks the position of the RAD21 target band.

Replicate 1

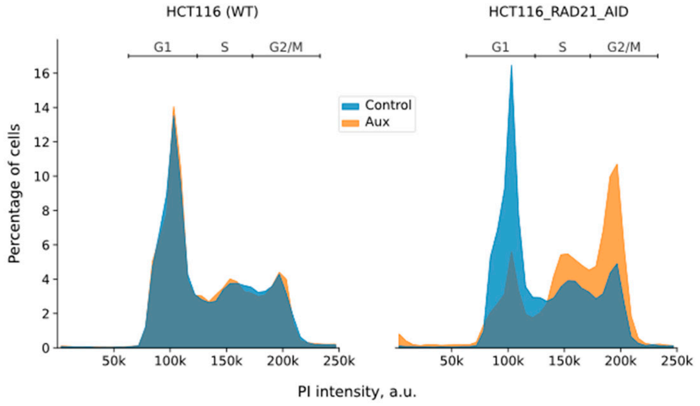

Cells	Treatment	G1, %	S, %	G2,%
HCT116 (WT)	Control	51.0	25.1	21.4
	Auxins	50.5	25.5	21.0
HCT116_RAD21_AID	Control	52.3	25.8	20.0
	Auxins	21.4	32.4	41.5

Replicate 2

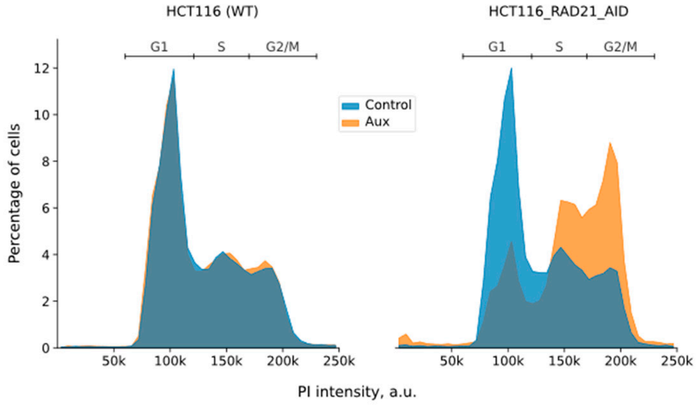

Cells	Treatment	G1, %	S, %	G2,%
HCT116 (WT)	Control	51.9	28.3	18.2
	Auxins	51.5	28.4	18.4
HCT116_RAD21_AID	Control	52.1	28.0	18.0
	Auxins	20.0	35.5	40.4

Replicate 3

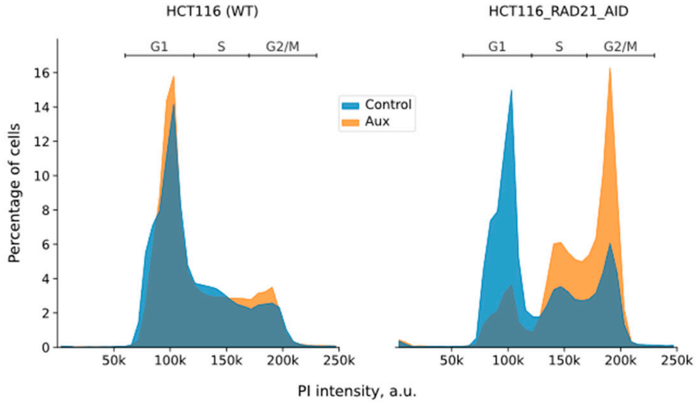

Cells	Treatment	G1, %	S, %	G2,%
HCT116 (WT)	Control	61.8	24.0	13.3
	Auxins	60.2	23.1	15.7
HCT116_RAD21_AID	Control	54.5	21.4	22.1
	Auxins	14.8	34.5	48.6

Figure S3. Cell cycle distribution profiles (propidium iodide staining) of HCT116 (WT) and HCT116_RAD21_AID cells in the absence (control) and presence of auxins (aux). The tables provide the percentage of cells in the indicated cell cycle phases. The results of three replicates are shown.

Replicate 1

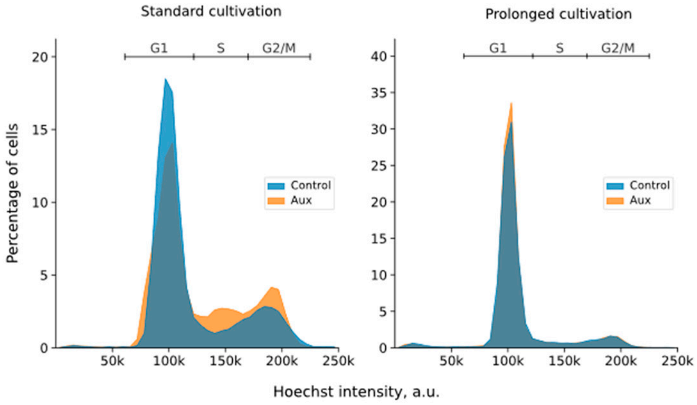

Cells	Treatment	G1, %	S, %	G2,%
Standard cultivation	Control	71.7	11.0	16.1
	Auxins	60.0	18.6	20.3
Prolonged cultivation	Control	84.2	5.8	7.4
	Auxins	85.1	5.4	7.4

Replicate 2

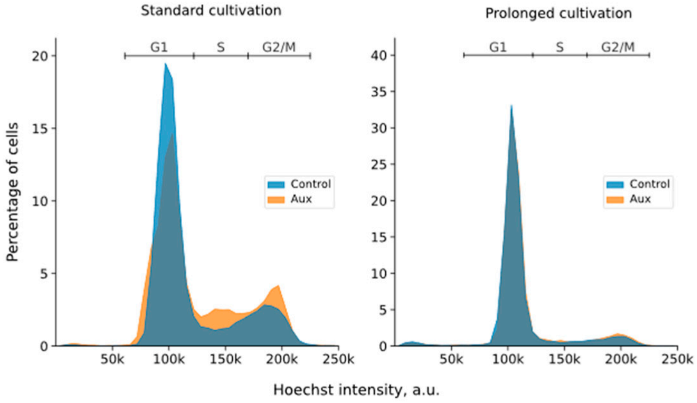

Cells	Treatment	G1, %	S, %	G2,%
Standard cultivation	Control	72.7	10.8	15.8
	Auxins	61.7	17.7	19.6
Prolonged cultivation	Control	84.6	5.5	7.6
	Auxins	83.4	5.8	8.8

Replicate 3

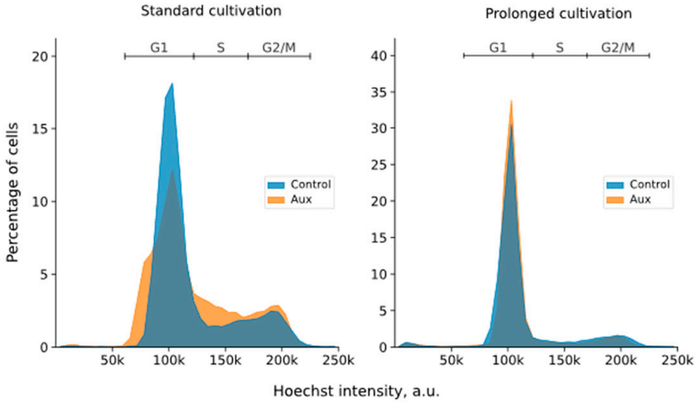

Cells	Treatment	G1, %	S, %	G2,%
Standard cultivation	Control	72.1	13.1	14.0
	Auxins	62.6	20.8	15.8
Prolonged cultivation	Control	81.2	6.2	9.6
	Auxins	85.5	4.9	7.4

Figure S4. Cell cycle distribution profiles (Hoechst33342 staining) of HCT116_RAD21_AID cells under standard culture conditions and prolonged culture conditions, in the absence (control) and presence of auxins (aux). The tables provide the percentage of cells in the indicated cell-cycle phases. The results of three replicates are shown.

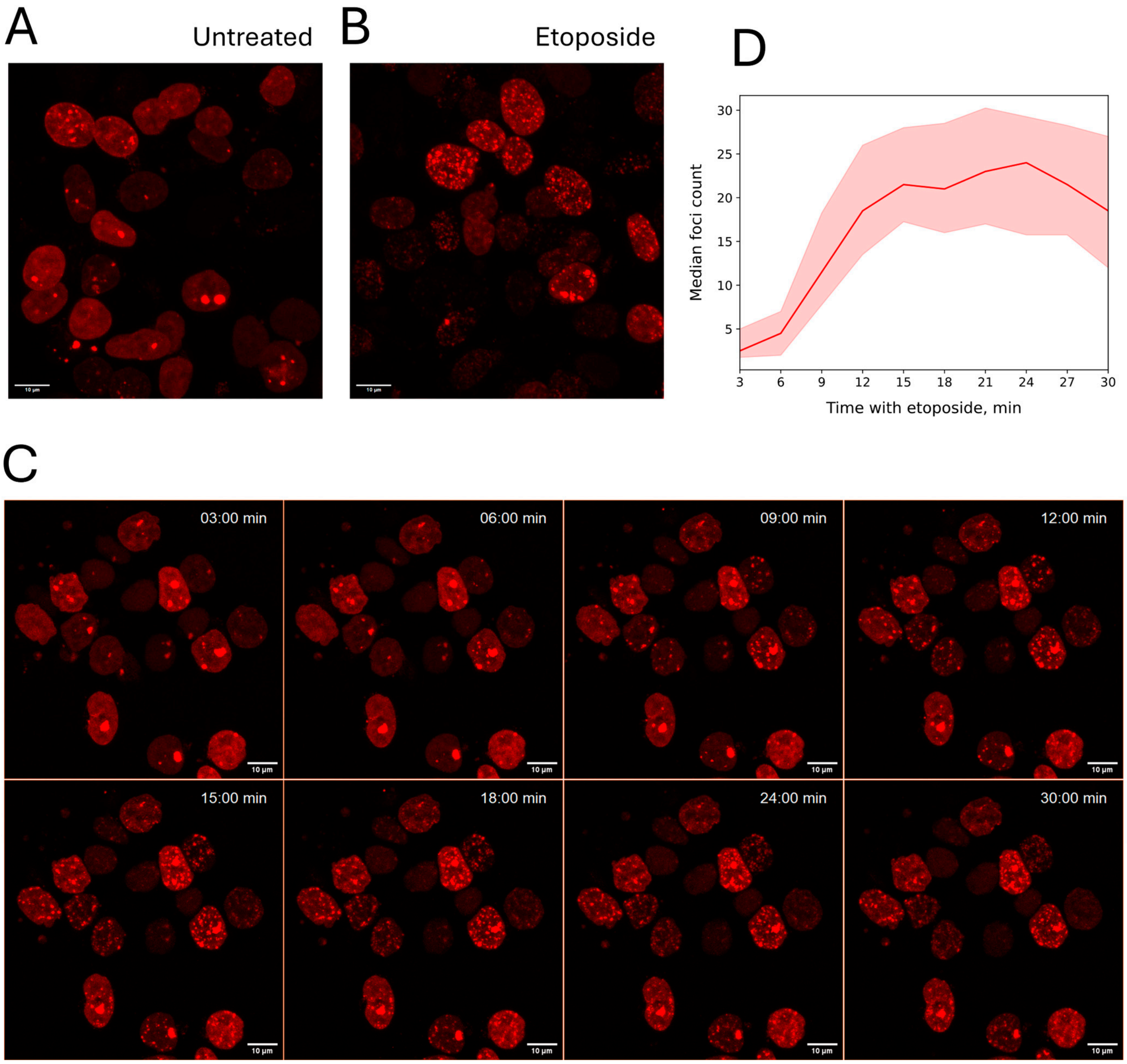

Figure S5. The FusionRed-BP1-2 reporter allows the visualization of etoposide-induced DSBs. (**A**) Untreated HCT116_RAD21_AID cells expressing the FusionRed-BP1-2 DSB reporter. (**B**) HCT116_RAD21_AID cells expressing the FusionRed-BP1-2 DSB reporter, treated with etoposide (100 µg/mL) for 20 min. (**C**) Time-lapse showing the DSB induction by etoposide (100 µg/mL). A time-lapse was recorded after etoposide addition (one frame/3 min). The first time point was 3 min after etoposide addition (the full time-lapse is included as **Supplementary video 2**). (**A–C**) show the maximum intensity Z-projections of the confocal images. (**D**) Kinetics of break induction by etoposide. The median number of FusionRed-BP1-2 fluorescence foci in cells and the interquartile range (IQR) are depicted. N = 40 cells.

Table S1. Sequences of utilized oligonucleotides and primers.

Name	Sequence
gRAD21_f	CACCGCAAGGTTCCATATTATATA
gRAD21_r	AAACTATATAATATGGAACCTTGC
mAID_SalI_link_f	ATATAGTCGACGGTGCAGGCGCCAAGGAGAAGAGTGCTTGTCCTAAAGA
mAID_BstV2I_KpnI	ATATAGGTACCGAAGACTACCGGTTTTATACATCCTCAAATCGAT-TTTCCTCAAGTA
T2A_Neo_f_AsiGI	TATATACCGGTGAGGGCAGAGGAAGTCTTCT
Neo_r_SacI	TATATGAGCTCTCAGAAGAACTCGTCAAGAAG
T2A_Hygro_f_AsiGI	TATATACCGGTGAGGGCAGAGGAAGTCTTCTAACATGCGGTGACGTGGAG-GAGAATCCCGGCCCTAAGCCTGAACTCACCGCGAC
Hygro_r_SacI	TATATGAGCTCCTATTCCTTTGCCCTCGGACGAG
HA_L_f	CAAGCTTGCATGCCTGCAGGTGTCTGCAAAATGCCAAGCA
HA_L_r	TCTCCTTGGCGCCTGCACCGCCTATAATATGGAACCTTGGTCCAGGTG
HA_R_Neo_f	TTCTTGACGAGTTCTTCTGAGAGCTATAAGGAGCTAGAAGCATTATAGCTAG
HA_R_Hygro_f	GTCCGAGGGCAAAGGAATAGGAGCTATAAGGAGCTAGAA-GCATTATAGCTAG
HA_R_r	CATGATTACGAATTCGAGCTCCAAACCAGGAGTGTGCAG
OsTIR_f_MluI	ATATAACGCGTGCAGGCTGGCGCCACCATGACATACTTTCCTGAAGAGGTCG
OsTIR_r_XbaI	ATATATCTAGATCACAGAATCTTCACAAAGTTGGGAG
BP1_Xho_f	AATAACTCGAGGGAAGCCAGGGAGAAGAAGAGTTTGA
BP1_Xba_r	AATAATCTAGATCATTCACCGGTGTTGTCTCCACT
RAD21_f	CCGGAAAAGCATGGAGGAGA
RAD21_r	GGGTATACACTGAAGTCTGAGTTTC
mAID_r	CCACCGCTTGATTTTTGGCA
Neo_f	GAAGGGACTGGCTGCTATTG
Hygro_f	GATCTTAGCCAGACGAGCGG
RAD21_f2	AGCGTGCTCTTGCTAAAACTGG
T2A_r	AGGGCCGGGATTCTCCT
OsTIR_f1	CCCAACCTGAGGTCTCTGCG
OsTIR_r1	CAGGGTTGGTGCATAGCTCA
